# Endoscopic Ultrasound‐guided Drainage With Lumen‐apposing Metal Stent versus Plastic Stent for the Treatment of Pancreatic Pseudocyst: A Systematic Review and Meta‐analysis

**DOI:** 10.1002/deo2.70165

**Published:** 2025-06-22

**Authors:** André Orsini Ardengh, Thiago Arantes de Carvalho Visconti, Marcelo Klotz Dall'Agnol, Ygor Rocha Fernandes, Matheus de Oliveira Veras, Evellin Souza Valentim dos Santos, Marcos Eduardo Lera do Santos, Sergio Eiji Matuguma, José Celso Ardengh, Wanderley Marques Bernardo, Eduardo Guimarães Hourneaux de Moura

**Affiliations:** ^1^ Gastroenterology Department, Gastrointestinal Endoscopy Unit, Hospital das Clínicas University of São Paulo (USP) Medical School São Paulo Brazil; ^2^ Digestive Endoscopy Service Hospital Moriah São Paulo Brazil; ^3^ Department of Surgery and Anatomy Digestive Endoscopy Section Hospital das Clínicas, Ribeirão Preto Medical School University of São Paulo (HCFMRP‐USP) Ribeirão Preto Brazil

**Keywords:** double pigtail plastic stents, endoscopic ultrasonography‐guided drainage, endosonography, lumen‐apposing metal stents, pancreatic pseudocyst

## Abstract

**Background:**

Pancreatic pseudocyst (PP), following acute or chronic pancreatitis, may become symptomatic or persist beyond 6–8 weeks, requiring drainage. Endoscopic ultrasonography‐guided drainage (EUS‐D) is the preferred method, using double pigtail plastic stents (DPPS) or self‐expandable metallic stents (SEMS), such as lumen‐apposing metal stents (LAMS). This meta‐analysis compares DPPS and LAMS in EUS‐D for PP, focusing on technical success, clinical success, adverse events (AEs), recurrence, and procedure time.

**Methods:**

A search strategy was conducted in MEDLINE, Embase, Lilacs, and Cochrane databases according to PRISMA guidelines. Random‐effect models were used for statistical analysis based on intention‐to‐treat. Heterogeneity was assessed using the I^2^ test. The risk of bias was assessed using the Risk of Bias in Non‐randomized Studies—of Exposures tool. The quality of evidence was assessed using the Grading of Recommendations Assessment, Development, and Evaluation Tool.

**Results:**

Ten studies were included: one prospective cohort and nine retrospective cohorts, conducted between 2014 and 2024. A total of 502 patients with PP were treated with EUS‐D. The clinical success rate was higher using LAMS (Risk ratio [RR] = 1.05; 95% confidence interval [CI]: 1.01; 1.09; I^2^ = 0%), with shorter procedure time (Mean difference = ‐16.30; 95% CI: ‐27.65; ‐4.94; I^2^ = 86%) compared to DPPS. No statistical difference was observed for early and late AEs, recurrence, or technical success.

**Conclusion:**

The study demonstrated that LAMS has a higher clinical success rate and a shorter procedure time compared to DPPS. There is no difference in terms of early and late AEs, recurrence, and technical success.

## 1 Background

Pancreatic fluid collection (PFC) is one of the complications following an episode of acute pancreatitis (AP). According to the Atlanta classification, PFCs are divided into four types: acute PFC, acute necrotic collection, pancreatic pseudocyst (PP), and walled‐off necrosis (WON) [1, 2]. Based on the time of evolution after the onset of the disease (>4 weeks) and the absence of necrosis, a PFC can be classified as a PP [1, 2, 3]. Commonly, PPs are asymptomatic, but if they present infection, gastrointestinal obstruction, abdominal pain, or persist without resolution for more than 6–8 weeks after the onset of AP, drainage is indicated to improve the outcomes for patients with symptomatic PP [1, 4, 5].

Endoscopic drainage (ED) is the preferred method of treatment, and endoscopic ultrasonography‐guided drainage (EUS‐D) is considered the first‐line treatment for PFCs (PP or WON) at major medical centers around the world [4, 6, 7, 8, 9, 10, 11]. EUS‐D, percutaneous‐guided drainage (PD), and minimally invasive surgery‐drainage (SD) are the most used therapeutic approaches for PFCs [12]. In cases where EUS‐D fails due to the distance between the PP and the upper gastrointestinal tract wall, making access impossible, PD is indicated. In contrast, PD may present as an adverse event (AE) the creation of a pancreatic‐cutaneous fistula, which can be difficult to resolve. In exceptional cases, SD can be used; however, it significantly increases costs and hospitalization time compared to EUS‐D and PD [4, 8, 9, 13, 14, 15].

During EUS‐D of PPs, two different types of stents can be used: double pigtail plastic stents (DPPS) and biliary self‐expandable metallic stents (SEMS). SEMS include biliary fully covered SEMS (FCSEMS) and those recently introduced, known as lumen‐apposing metal stents (LAMS). Although LAMS are specifically designed for EUS‐D of any PFC (WON and PP) and offer technical advantages, there are no robust and definitive recommendations regarding the ideal type of stent for treating PP [6, 16]. Furthermore, many published studies have included both WON and PP, providing overall results for the two types of collections without performing subgroup analyses. It is expected that WON and PP may present different outcomes after EUS‐D, as several factors could influence the results, such as the fluidity of the liquid, presence or absence of necrosis, size, and location of the PP. Therefore, the authors conducted this meta‐analysis to resolve conflicting results in light of current knowledge.

The objective of this systematic review and meta‐analysis is to compare the use of DPPS versus LAMS in patients with PP undergoing EUS‐D. We highlighted technical success, clinical outcomes, occurrence of AEs, and recurrence, to determine the best stent to use in patients with PP.

## 2 Methods

### 2.1 Protocol and Registration

This study was performed according to PRISMA guidelines (Preferred Reporting Items for Systematic Reviews and Meta‐Analysis) and registered in PROSPERO (International Prospective Register of Systematic Reviews) under the register CRD42024574507 [17].

### 2.2 Search Strategy

The search was conducted in LILACS (Latin American and Caribbean Health Science Information Database); IBECS (Indice Bibliográfico Español de Ciencias de la Salud; MEDLINE via Ovid (1966 to present); EMBASE via Elsevier (1974 to present) and CENTRAL (Cochrane Central Register of Controlled Trials) in the Cochrane Library (2020) with simplified strategies for each database (Table S1).

### 2.3 Eligibility Criteria

‐ Type of studies: comparatives studies (with parallel‐group design) evaluating the performance of lumen‐apposing metallic stents compared to double pig‐tail plastic stents on the treatment of PP. We did not impose language, publication date, or status restrictions for potential retrieved records.
‐ Types of participants: Adult patients aged > 18 years old with PP classified according to the revised 2012 Atlanta classification of AP [1].
‐ Exclusion criteria: studies that provide overall results in the comparison of endoscopic‐ultrasonography‐guided drainage of PFC that do not differentiate between the results of drainage for WON and PP. It also excluded studies that did not differentiate between the types of SEMS (LAMS and FCSEMS), as the intention of the study is to analyze only the effect of LAMS on PP. In addition, studies that compared transmural drainage to transpapillary drainage were excluded.

## 3 Data Collection and Analysis

### 3.1 Selection of Studies

Two review authors (André Orsini Ardengh and Thiago Arantes de Carvalho Visconti) screened titles and abstracts after removing duplicates and identified potentially eligible studies according to our eligibility criteria from retrieved records of defined databases. After this phase, full text was acquired and assessed once more the eligibility criteria of these studies for inclusion, providing reasons for the excluded studies. Lastly, we merged published reports from the same study. The entire screening process was carried out on Rayyan software [18].

### 3.2 Data Extraction and Management

Data extraction and management were performed using a data extraction form in an Excel spreadsheet. Methods data (study design and analysis), data on the characteristics of the participants (country, total number of participants, age, sex, inclusion, and exclusion criteria), interventions (therapy type), and outcome measures were extracted. In case of disagreements, any discrepancies were resolved by consensus after retrieving the information from the original article. When it was needed, we contacted the authors of primary studies for further information.

### 3.3 Type of Outcome Measures

#### 3.3.1 Primary Outcomes

The primary endpoint was clinical success, defined by the resolution of symptoms, or the resolution of the PP evidenced during follow‐up after the EUS‐D.

#### 3.3.2 Secondary Outcomes

1 ‐ Technical success as effective transgastric or transduodenal stent placement during endoscopic procedure.
2 ‐ Adverse events (early or late) as the complications related to the endoscopic procedure.
3‐ Procedure duration is the time between the passage of the echoendoscope to carry out transmural drainage until stent deployment.
4‐ Pancreatic pseudocyst recurrence, defined as recurrent symptoms and increase in PP diameter.

### 3.4 Assessment of Risk of Bias in Included Studies

Two authors (André Orsini Ardengh and Thiago Arantes de Carvalho Visconti) assessed the risk of bias of each included study using a tool named “Risk of Bias in Non‐randomized Studies—of Exposures (ROBINS‐E)” for observational studies according to the recommendations in the Cochrane Handbook for Systematic Reviews of Interventions [19]. The following definitions were used in the assessment of the risk of bias: due to confounding, arising from the measurement of the exposure, in the selection of participants into the study (or into the analysis), due to post‐exposure interventions, due to missing data, arising from the measurement of the outcome, in the selection of the reported result. For signaling questions within each domain for each outcome, one of the five possible answers was provided in each tool (“Yes”, “Probably yes”, “No”, “Probably no” and “No information”), judging as “Low risk of bias”, “Some concerns” or “High risk of bias”. According to the algorithm result, the overall risk‐of‐bias judgment for each outcome was the least favorable assessment across the domains. When it was needed, we contacted the authors of primary studies for further information. Findings were summarized in the “Risk of Bias” tables and figures.

### 3.5 Statistical Analysis

Statistical analysis was performed according to the statistical recommendations described in the Cochrane Handbook for Systematic Reviews of Interventions. We compiled risk ratios (for dichotomous outcomes) and mean differences (for continuous outcomes) with 95% confidence interval (CI) of individual studies using a random‐effects meta‐analysis (when results of two or more similar studies could be pooled). An intention‐to‐treat analysis was used as far as possible. Data was analyzed using meta and metasens packages implemented on R program and the results of the meta‐analysis were presented as a forest plot.

The quality of the evidence was graded as high, moderate, low, and very low according to the Grading of Recommendations Assessment, Development, and Evaluation (GRADE) tool [20].

### 3.6 Assessment of Heterogeneity

Statistical heterogeneity was assessed employing the Cochran Q test to determine the strength of evidence that heterogeneity is genuine. We considered a threshold of *p*‐value < 0.1 as an indicator of the presence of heterogeneity (genuine variation in effect sizes). In addition, we performed by examining the I^2^ statistic interpreting as follows: <25% (no heterogeneity); 25%–49% (low heterogeneity); 50%–74% (moderate heterogeneity); ≥75% (high heterogeneity).

### 3.7 Assessment of Reporting Biases

Publication bias was assessed for those outcomes that showed a relevant statistical difference, whenever possible, and for outcomes with more than 10 studies included in the analysis, by inspecting funnel plots and performing Egger's test.

### 3.8 Sensitivity Analysis

A sensitivity analysis was conducted by excluding studies with a high risk of bias to assess the influence of these studies on the observed outcomes. If the results remain unchanged despite the exclusion of these studies, it will strengthen the findings.

## 4 Results

### 4.1 Bibliographical Research

**FIGURE 1:**
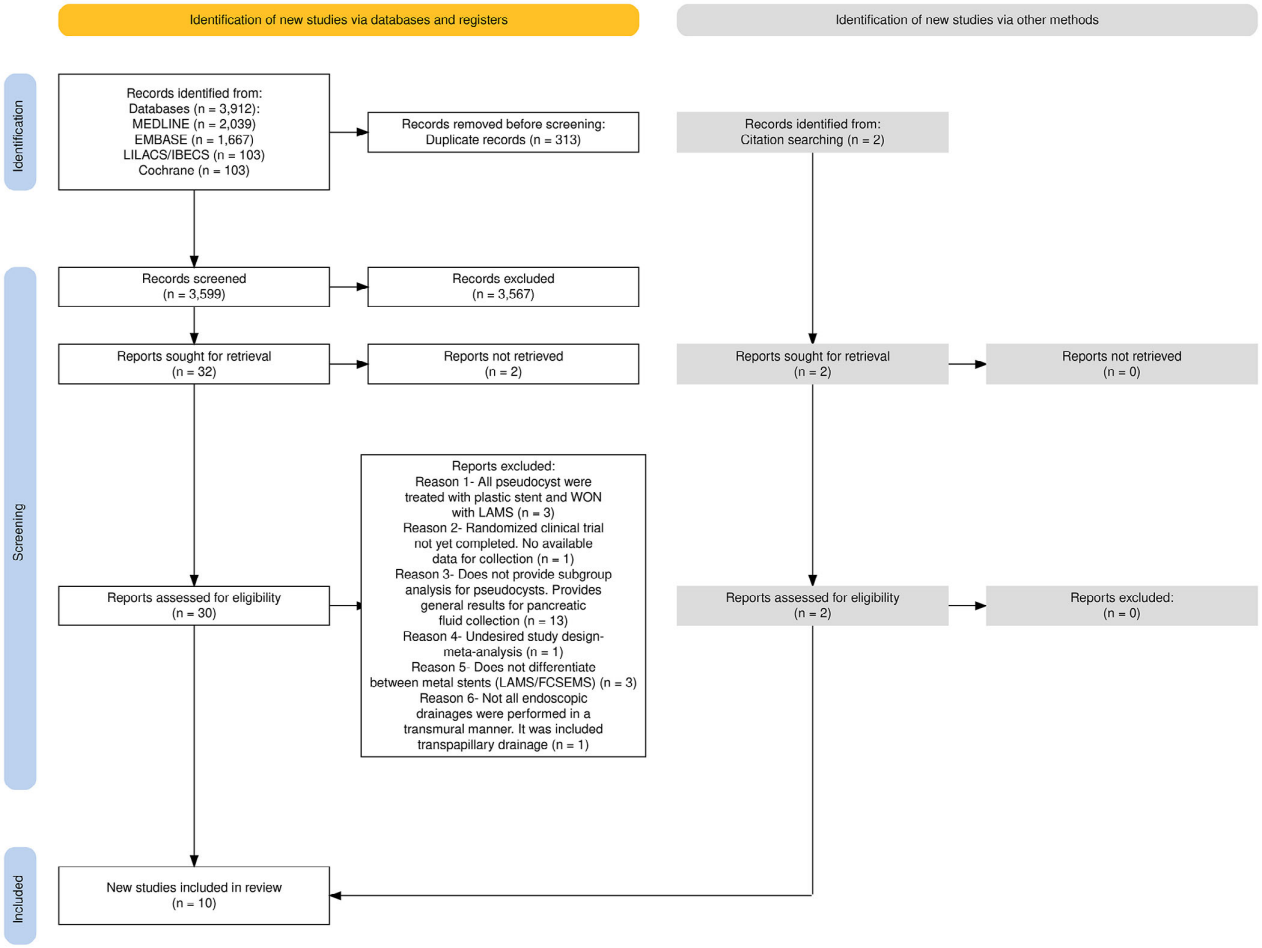
Prisma flow diagram of included and excluded studies.

The initial search strategy identified a total of 3912 studies. Of these, 313 duplicates were excluded. After reading the titles and abstracts, 30 studies were selected to meet the inclusion and exclusion criteria. A total of 8 studies met the criteria and were selected for the final analysis. In addition, two more studies were selected from citation searching and met the inclusion and exclusion criteria. The schematic diagram of the identification and selection of the 10 [6, 21, 22, 23, 24, 25, 26, 27, 28, 29] studies analyzed is shown in Figure 1.

### 4.2 Study Characteristics

Of 10 [6, 21, 22, 23, 24, 25, 26, 27, 28, 29] studies included in this meta‐analysis, one was a prospective cohort (Khodakaram et al.) [21] and nine were retrospective cohorts. The studies were conducted between 2014 and 2024, in different countries around the world. Some studies, such as those by Yang et al. [6] and Gkolfakis et al. [22], were multicenter studies conducted in more than one country. A total of 502 patients with PP were included and treated with EUS‐D: 305 were assigned to the DPPS group and 197 to the LAMS group. Characteristics of the study are shown in Table 1.

**TABLE 1:** Studies characteristics.

Study	Year	Study design	Country	Number of patients	Characteristics	Outcomes analyzed
**Khodakaram K**	2024	Prospective	Sweden	37	Median age was 57 (range: 21–92) years. Sex: 38% female; 62% male. DPPS used: 2 plastic stents‐7Fr × 7 cm (100%). LAMS 15 and 20 mm diameter.	Technical success, Clinical success (defined as a complete resolution of the PFC on CT with the initial stent and within 1 month), and early adverse events
**Gkolfakis P**	2022	Retrospective	Greece; Italy; Croatia and Hungary.	92	The median age was 56.2 ± 12.7 years. Sex: 25% female; 75% male. DPPS used: 1 plastic stent‐ 65%; 2 plastic stents‐ 35%; 7 Fr x 3 cm – 67.5% and 9Fr x 3cm – 7.5%. LAMS used: Diameter 10 mm – 25% and 15 mm – 75%. PP location: Head 14.1%; Istmus 5.5%; Body 43.5%; Tail 36.9%. PP size: 11.8±4.7 cm	Technical success, Clinical success (defined as a decrease in the size of the PFC to ≤3 cm on cross‐sectional imaging, with resolution of symptoms at 6‐month follow‐up), and early adverse events
**Kayal A**	2021	Retrospective	Canada	15	Median age was 54.0 (38–64.8) years. Sex: 33.3% female; 66.7% male. DPPS used: one or two 7–9 cm (length) × 10 Fr LAMS used: 10 × 15 mm (diameter)‐ 100%. PP Location: Head 33.3%; Tail/body 60%; Unclear/Peripancreatic 6.7%. PP size: 13 (8.5–16) cm. Cause of initial pancreatitis: Gallstones 26.7%; Alcohol 40%; Trauma 6.7%; Idiopathic 13.2%; Drug‐related 6.7%; Unknown 6.7%	Technical success, Clinical success (defined as complete collection resolution or significant size reduction of ≥ 50%, with resolution of a patient's symptoms and no need for reintervention at 3 months), early adverse events, late adverse events, and recurrence
**Al Lehibi A**	2021	Retrospective	Saudi Arabia	16	The median age was 43.5 ± 19 years. Sex: 31% female; 69% male. Drainage approach: transgastric 100%. DPPS 10 Fr × 7–10 cm in 36.4% LAMS 10 mm × 15 mm and 20 mm × 16 mm in 57.6%.	Clinical success (defined by the resolution of symptoms, or the resolution of the PFC on imaging during the 4 weeks) and early adverse events
**Yang J**	2019	Retrospective	USA, Italy, and Germany	205	Median age was 54.5 [SD 14.1] years. Sex: 36.6% female; 63.4% male. PP location: Head 22.9%; Body 44.4%; Tail 27.8%; Unclear/peripancreatic: 4.9%. PP Size: 10.18 (5.36) cm treated with DPPS and 10.64 (4.88) cm treated with LAMS. Cause of initial pancreatitis: Gallstones 20%; Alcohol 32.2%; Trauma 5.9%; Idiopathic 19.5%; Autoimmune 0.5%; Post‐ERCP 3.4%; Unknown 18.5%. Drainage approach: transgastric 85.9%; Transduodenal 13.6%; Others 0.5% Total diameter of all stents LAMS: 15 mm (10–15) and DPPS 6 mm (5–10).	Technical success, Clinical success (defined as a reduction of the pancreatic pseudocyst to ≤ 3 cm on CT/MRI, with corresponding clinical symptom resolution within 6 months of stent insertion, and without the need for percutaneous drainage or surgery), early adverse events, time of procedure and recurrence
**Shin HC**	2019	Retrospective	Korea	25	Median age was 56 (23–86) years. Sex: 20% female; 80% male. PP size: 8.08 (4.3–19.9). Size and number of stents not available (NA).	Technical success, Clinical success (defined by resolution of symptoms in combination with a decrease in the PFC size (complete resolution or reduction in size >75% or <2 cm) on follow‐up Imaging), early adverse events, and time of procedure
**Cho CM**	2018	Retrospective	South Korea	27	The median age was 54 years. Size and number of stents not available.	Technical success and recurrence
**Ge N**	2017	Retrospective	China	52	The median age was 50.35 ± 12.71. Sex: 44.2% female; 55.8% male. PP Size: 9.53 ± 4.49. Single DPPS: 10Fr LAMS 10 mm/35 mm. In some cases, a DPPS 10 Fr/7 cm, 10 Fr/3 cm, or 10 Fr/5 cm was introduced through the metal stent for auxiliary drainage.	Technical success, Clinical success (resolution of clinical symptoms in combination with a decrease in the size of the PPs to 3 cm on imaging within 3 months), early adverse events, and late adverse events
**Bang JY**	2017	Retrospective	USA	21	Median age was 52.9 (18.4) years. Sex: 40% female/ 60% male. DPPS group and 50.7 (15.4) in the LAMS group. DPPS used: Two plastic stents – 100%; 7 Fr – 100%. LAMS used: Diameter 15 mm – 100%	Technical success, Clinical success (defined as resolution of PFC to ≤2 cm on CT/MRI in association with clinical resolution of symptoms at 8‐week follow‐up), and late adverse events
**Mukai S**	2014	Retrospective	Japan	12	NA	Technical success, Clinical success (definition not available), and early adverse events

Abbreviations: CT: computed tomography; DPPS: double pigtail plastic stents; LAMS: lumen‐apposing metal stents; MRI: magnetic resonance imaging; NA: not available; PFC: pancreatic fluid collections; PP: pancreatic pseudocyst.

### 4.3 Risk of Bias and Quality of Studies

Application of the ROBINS‐E tool revealed that the observational studies by Mukai et al.  [29] and Cho et al. [26] have an overall “high risk of bias” resulting from confounding bias and “some concerns” about bias of selection and missing data. All other studies revealed “some concerns” regarding the risk of bias in the selection domain and some of them on missing data (Table S2).

## 5 Meta‐analysis

### 5.1 Technical Success

**FIGURE 2:**
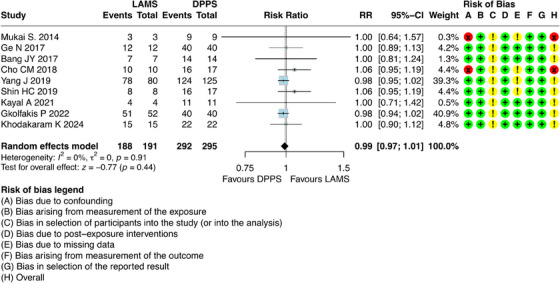
Technical success of double pigtail plastic stents (DPPS) versus lumen‐apposing metal stents (LAMS) for the treatment of pseudocysts.

For this outcome, it was possible to combine results from nine studies [6, 21, 22, 23, 25, 26, 27, 28, 29], which involved 486 participants with DPPS (*n =* 295) and LAMS (*n =* 191). The absolute number of technical successes was 188 out of 191 (98.42%) patients in the LAMS group and 292 out of 295 (98.98%) in the DPPS group. The results showed that there was no difference regarding the technical success between DPPS and LAMS (risk ratio [RR] = 0.99; 95% CI: 0.97; 1.01; I^2^ = 0%) (Figure 2).

### 5.2 Clinical Success

**FIGURE 3:**
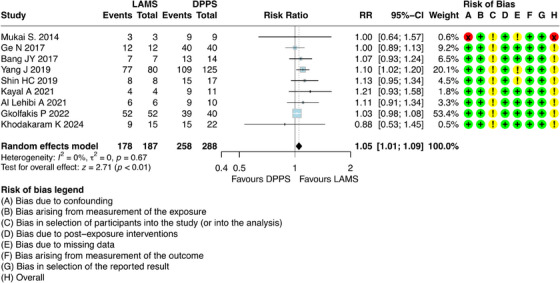
Clinical success of double pigtail plastic stents (DPPS) versus lumen‐apposing metal stents (LAMS) for the treatment of pseudocysts.

For this outcome, it was possible to combine results from nine studies [6, 21, 22, 23, 24, 25, 27, 28, 29], which involved 475 participants with DPPS (*n =* 288) and LAMS (*n =* 187). The results regarding this outcome showed that patients who underwent ultrasound‐guided drainage with LAMS had a higher clinical success compared to DPPS [RR = 1.05; 95% CI: 1.01; 1.09; I^2^ = 0%] (Figure 3).

### 5.3 Early Adverse Events

**FIGURE 4:**
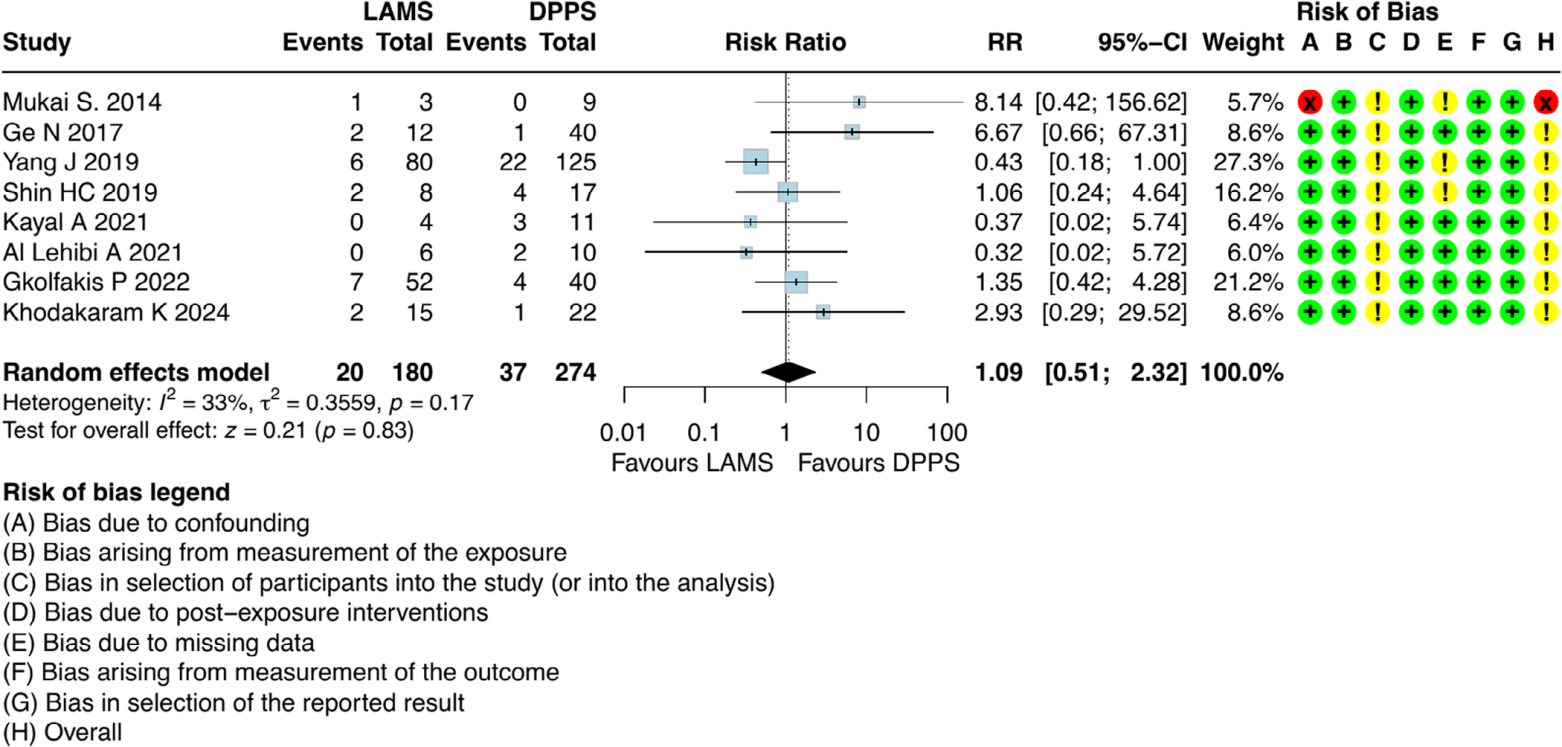
Early adverse events of double pigtail plastic stents (DPPS) versus lumen‐apposing metal stents (LAMS) on the treatment of pseudocysts.

For this outcome, it was possible to combine results from eight studies [6, 21, 22, 23, 24, 25, 27, 29], which involved 454 participants with DPPS (*n =* 274) and LAMS (*n =* 180). The results showed that there was no difference regarding early AEs between DPPS and LAMS [RR = 1.09; 95% CI: 0.51; 2.32; I^2^ = 33%] (Figure 4).

The evaluation of early AEs did not reveal a superiority of one technique over the other, with rates of 11.1% for the LAMS group and 13.5% for the DPPS group. The AEs observed in the studies reviewed included: bleeding, perforation/migration, cyst leakage, pain, and infection.

The most frequently observed early AEs were infections and bleeding with a total of 16 bleeding events recorded, and 13 of them occurring in the DPPS group. Additionally, there were 12 cases of infection in total, and 11 of them were also associated with the DPPS group (Table S3). However, there was no statistically significant difference between the two methods regarding bleeding, infection, migration/perforation, and cyst leak (Table S4).

### 5.4 Late Adverse Events

**FIGURE 5:**
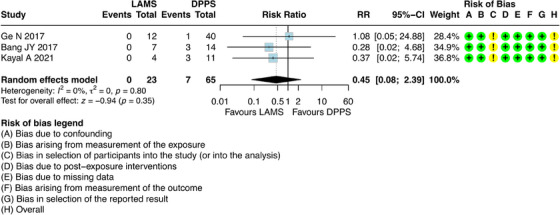
Late adverse events of double pigtail plastic stents (DPPS) versus lumen‐apposing metal stents (LAMS) on the treatment of pseudocysts.

For this outcome, it was possible to combine results from three studies [23, 27, 28], which involved 88 participants with DPPS (*n =* 65) and LAMS (*n =* 23). The results showed that there was no difference regarding late AEs between DPPS and LAMS [RR = 0.45; 95% CI: 0.08; 2.39; I^2^ = 0%] (Figure 5).

DPPS exhibited a rate of 10.76%, whereas no late AEs were reported in the LAMS group. Among the late AEs in the DPPS group, migration/perforation occurred in three patients, infection in three patients, and abscess formation in one (Table S5).

### 5.5 Recurrence of Pancreatic Pseudocyst

For this outcome, it was possible to combine results from three studies [6, 23, 26], which involved 247 participants with DPPS (*n =* 153) and LAMS (*n =* 94).

Initially, intention‐to‐treat (ITT) analysis was performed. ITT analysis is fundamental regardless of whether they received or completed the assigned treatment, or if there was loss to follow‐up. This method minimizes selection bias and provides a more conservative and clinically relevant estimate of the treatment effect under “real‐world” conditions, reflecting clinical practice where patients may not adhere perfectly to the protocol.

**FIGURE 6:**
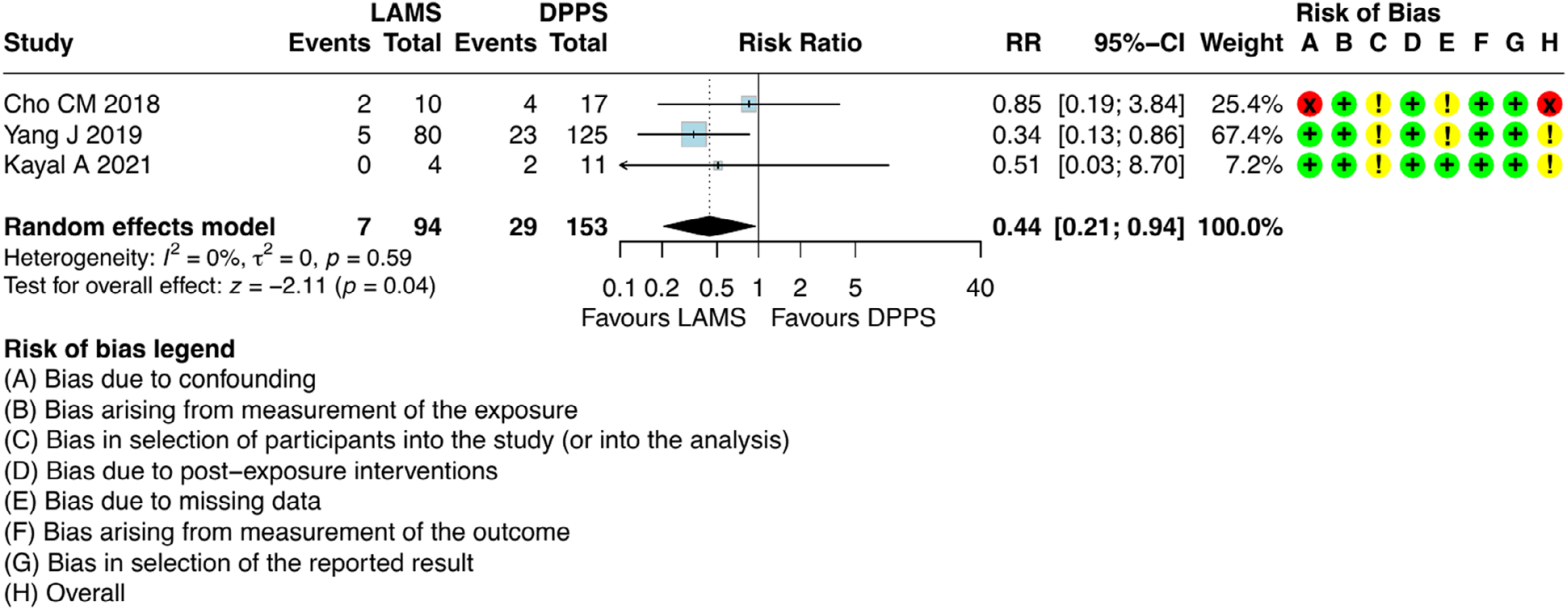
Recurrence after endoscopic ultrasonography‐guided drainage (EUS‐D) with double pigtail plastic stents (DPPS) versus lumen‐apposing metal stents (LAMS) on the treatment of PPs.

The results regarding recurrence, when analyzed according to the ITT, showed that there was a favorable outcome for those patients who underwent EUS‐D with LAMS, with a higher recurrence in patients who underwent drainage with DPPS. [RR = 0.44; 95% CI: 0.21; 0.94; I^2^ = 0%] (Figure 6).

However, it is crucial to note that there was a significant loss of follow‐up (36%) in the studies addressed in this analysis. While ITT analysis is robust against exclusion bias, a high rate of missing data can compromise both the precision and validity of its estimates. Significant loss to follow‐up may dilute the observed treatment effect and introduce uncertainty, particularly if the reasons for missing data are not randomly distributed between groups. In scenarios with substantial loss to follow‐up, the assumptions of ITT analysis may be weakened, making the results somewhat unreliable.

Therefore, to provide a complementary and more robust assessment of efficacy under ideal conditions of adherence and complete follow‐up, a per‐protocol (PP) analysis was conducted. PP analysis includes only participants who strictly adhered to the study protocol, received the planned intervention, and completed the entire proposed follow‐up period.

**FIGURE 7:**
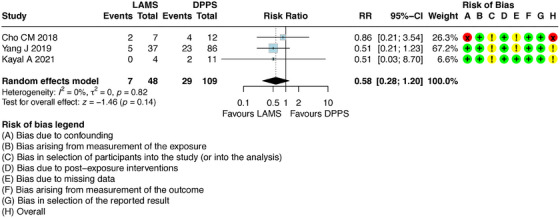
Per‐protocol study of pseudocyst recurrence.

This new PP analysis modified the result previously described by the ITT analysis. The PP analysis demonstrated that there is no difference in recurrence between the two methods with RR 0.58 [95% CI: 0.28–1.20] (Figure 7).

### 5.6 Procedure Time

**FIGURE 8:**
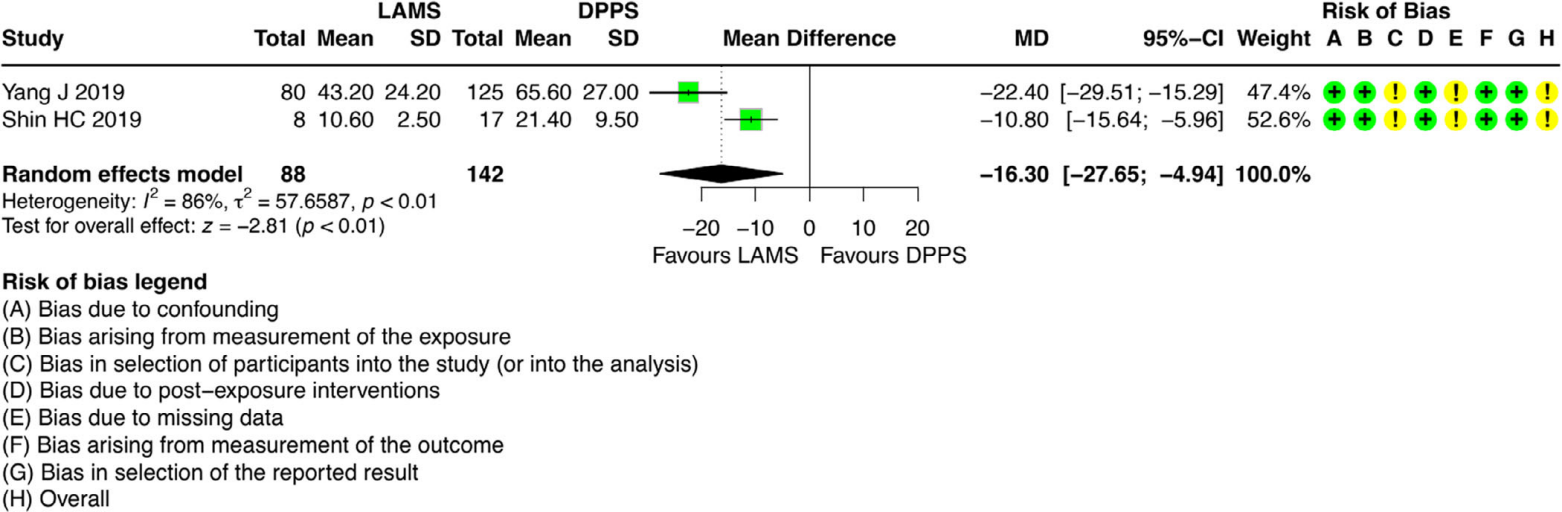
Procedure time of double pigtail plastic stents (DPPS) versus lumen‐apposing metal stents (LAMS) on the treatment of pseudocysts.

For this outcome, it was possible to combine results from two studies [6, 25], which involved 230 participants with DPPS (*n =* 142) and LAMS (*n =* 88). The results regarding this outcome showed that patients who underwent ultrasound‐guided drainage with LAMS had a shorter procedure time (minutes) compared to DPPS (Mean difference = ‐16.30; 95% CI: ‐27.65; ‐4.94; I^2^ = 86%) (Figure 8).

## 6 Sensitivity Analysis

### 6.1 Technical Success

Although two studies presented a high risk of bias for the confounding domain, we deemed it unnecessary to conduct a sensitivity analysis for this outcome, as the contribution of these studies to the combined estimate is exceedingly small (Cho et al. [26] = 4.4%; Mukai et al. [29] = 0.3%). Moreover, it is observed that the combined estimate is not significant, with RR = 1.01 [95% CI: 0.99 to 1.03].

### 6.2 Clinical Success

A study (Mukai et al. [29]) showed a high risk of bias due to confounding bias in the analysis. When this study was excluded from the analysis, the effect in favor of LAMS remained with RR = 1.05 [95% CI: 1.01–1.09] (Figure S1).

### 6.3 Early Adverse Events

For this outcome, one study (Mukai et al. [29]) presented a high risk of bias for the confounding domain, along with some concerns about participant selection in the analysis and the absence of data. Upon removing this study from the analysis, it is observed that the combined effect does not alter our conclusion, with RR = 0.93 [95% CI: 0.45–1.93]. After removing this study, heterogeneity decreased from I^2^ = 33% to I^2^ = 27% (Figure S2).

### 6.4 Late Adverse Events and Procedure Time

No sensitivity analyses were conducted for these outcomes, as the studies included in the analysis did not present a high risk of bias.

### 6.5 Recurrence of Pseudocyst

For recurrence, one study (Cho et al. [26]) presented a high risk of bias for the confounding domain and raised some concerns about selection bias and missing data. When this study was removed from the analysis, it was observed that the recurrence rate remained higher for DPPS with RR: 0.35 [95% CI: 0.15–0.85] compared to LAMS when ITT was analyzed (Figure S3).

## 7 Publication Bias (Small Studies Effect)

We planned to assess whether small‐study effects influenced any outcome with a statistically significant difference. However, this analysis was only possible for the clinical success outcome, which included nine studies—considered sufficiently close to the recommended threshold. As a result of the publication bias assessment, Figure S4 showed no evidence of publication bias, as demonstrated by Egger's test (*β* = 0.379; *p* = 0.465). For the procedure time outcome, publication bias was not assessed because only 2 studies were available, which is insufficient for this type of analysis.

## 8 Grading of Recommendations Assessment, Development, and Evaluation

**FIGURE 9:**
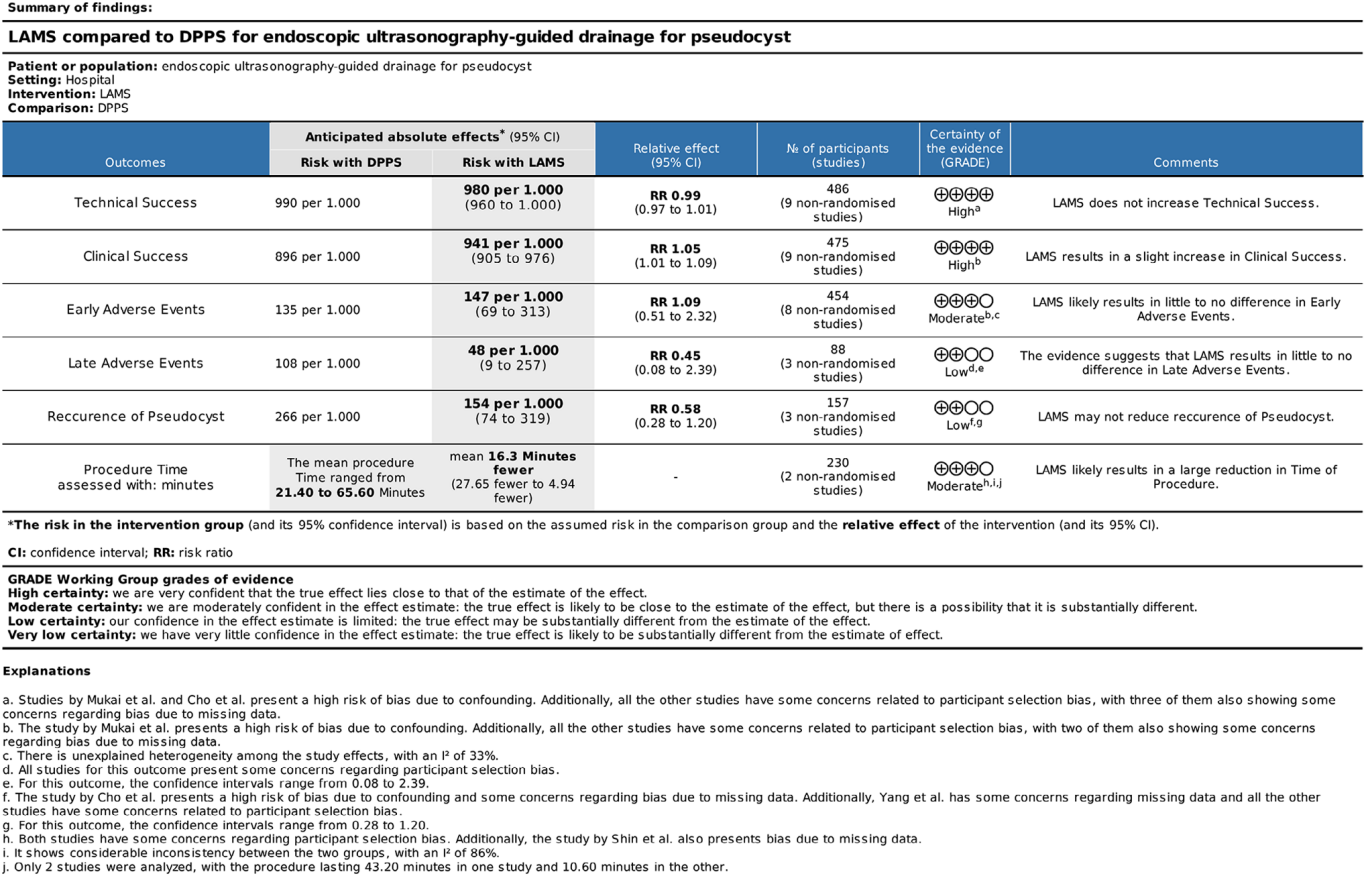
Grading of recommendations assessment, development, and evaluation (GRADE) tool for study outcomes. Legend: PP: Pancreatic pseudocyst; DPPS: Double pigtail plastic stents; LAMS: lumen‐apposing metal stents; EUS‐D: Endoscopic ultrasonography‐guided drainage.

After a detailed analysis of the outcomes, using the GRADE tool, regarding clinical success and technical success, the GRADE indicated a high level of certainty of evidence. For the outcomes of early AEs and time of procedure, the GRADE indicated a moderate level of certainty of evidence. However, for the outcomes of late AEs and recurrence, GRADE indicated a low level of certainty of evidence (Figure 9).

## 9 Discussion

Based on this analysis, EUS‐D with LAMS demonstrates higher clinical success and a shorter procedure time compared to DPPS, while maintaining similar rates of early and late AEs, technical success, and recurrence.

When outcomes are evaluated separately, technical success—defined as successful transgastric or transduodenal stent placement—was similarly high in both groups: 98.42% for LAMS and 98.98% for DPPS. This aligns with previous literature, including the 2021 meta‐analysis by Guzmán‐Calderón et al. [30], which supports the high efficacy of both techniques.

The clinical success outcome, defined as a symptom or PP resolution during follow‐up after EUS‐D, was superior with LAMS (95.18%) compared to DPPS (89.58%), with no detected heterogeneity among studies. This finding differs from Guzmán‐Calderón et al. [30], which found no statistical difference between the two methods. This may have occurred, as this meta‐analysis included a larger number of studies and patients analyzed regarding this outcome compared to Guzmán‐Calderón et al. [30]. Although DPPS success rates remain high, ranging from 85% to 95% [6, 31, 32, 33, 34, 35], due to the relatively fluid and debris‐free nature of the cyst content, this stent type presents specific limitations in comparison to LAMS. These limitations include a reduced lumen diameter, making necessary the use of multiple stents to achieve adequate drainage, and a propensity for stent occlusion, which is not unusual [36]. In contrast, LAMS provides a larger drainage diameter (approximately 1.5 cm), enabling more efficient and rapid fluid evacuation.

Adverse events assessment revealed no superiority between techniques. Both the present analysis and previous studies report a higher incidence of infection with DPPS, possibly attributable to occlusion by food debris and bacterial biofilm formation [6] (Table S2). Notably, a higher rate of bleeding was observed with DPPS, diverging from earlier findings, largely due to the influence of Yang et al. [6] (27.3%), where DPPS was associated with increased bleeding. This discrepancy may be explained by variations in AE interpretation, differences in terminology, and procedural complexity among endoscopists, even when applying the ASGE lexicon [6, 37, 38].

Recent concerns have been raised regarding an elevated risk of bleeding with LAMS in PFC drainage, likely due to rapid collapse of the collection resulting in vascular erosion or direct stent impingement, potentially leading to pseudoaneurysm formation and hemorrhage. The etiology of DPPS‐associated bleeding remains less well‐defined, as the literature is limited and lacks comprehensive data. However, general principles of ED procedures suggest potential mechanisms such as mechanical trauma from stent curvature, vascular injury during balloon dilation, erosion of the contralateral gastric or duodenal wall from excessively long stents, and complications related to stent migration [39].

In terms of PP recurrence (analyzed by ITT), recurrence was higher among patients treated with DPPS. However, interpretation is limited by factors such as short follow‐up durations (<6 months) in the studies by Kayal et al. [23] and Cho et al. [23, 26], and the loss of follow‐up of some patients in the studies by Yang et al. [6, 26] and Cho et al. [26] before completing 6 months, which reduces the strength of the evidence observed in the meta‐analysis. Moreover, some studies did not specify which patients had their LAMS removed and replaced with a pigtail, and which ones did not retain the pigtail after the LAMS drainage, which may introduce bias favoring metallic stents.

Therefore, a PP sensitivity analysis was conducted, focusing exclusively on patients who completed the follow‐up period. This analysis demonstrates no difference between the two methods, due to higher loss to follow‐up in the DPPS group. However, due to approximately 36% of patients lost, the evidence strength for this outcome is limited, and no definitive conclusions can be drawn.

Procedure time was significantly reduced with LAMS, as this approach typically requires a single stent and guidewire, usually without the need for balloon dilation as needed for DPPS placement. This difference may be even more pronounced when Hot‐LAMS systems are used. However, despite the technical ease of LAMS, some studies, such as Bang et al. [28], have shown higher overall hospital costs for the LAMS group (USD 58,649/nLAMS = 20 vs. USD 18,996/nDPPS = 40—p = 0.030), which is a drawback for device's accessibility and availability in lower‐complexity centers.

After this brief discussion, it is evident that this study has several limitations, particularly regarding the design of the studies included in this meta‐analysis. Since all reviewed studies were observational, with only one being prospective (Khodakaram et al. [21]), there is an increased risk of selection bias among participants. Another limitation is the significant loss of follow‐up in the recurrence outcome analysis and the insufficient data from studies pertaining to whether a plastic stent is placed after metallic stent removal preventing reliable conclusions about recurrence. Despite the aforementioned limitations, no randomized clinical trials specifically addressing PP drainage—without including WON drainage in their analysis—have been published, making the studies in this review the highest level of evidence currently available in the literature.

## 10 Conclusion

The study demonstrated that LAMS has a higher clinical success rate, and a shorter procedure time compared to DPPS. There is no difference in terms of early and late AEs, recurrence, and technical success.

## 11 Declaration of Generative Artificial Intelligence and Artificial Intelligence‐assisted Technologies in the Writing Process Statement

During the preparation of this work, the author(s) used Rayyan software in order to detect duplicate records during database searching. After using this tool/service, the author(s) reviewed and edited the content as needed and take(s) full responsibility for the content of the publication.

## Ethics Statement

Approval of the research protocol by an Institutional Reviewer Board: N/A

## Consent

N/A

## Conflicts of Interest

The authors declare no conflicts of interest.

## Clinical Trial Registration

Registered in PROSPERO (International Prospective Register of Systematic Reviews) under the register CRD42024574507.

## Supporting information




**Table S1**. Full search strategy for each database
**Table S2**. Risk of bias and quality of studies
**Table S3**. Types of early adverse events of DPPS versus LAMS on the treatment of pseudocysts
**Table S4**. Comparison between the two methods (LAMS and DPPS) for each type of early adverse event
**Table S5**. Types of late adverse events of DPPS versus LAMS on the treatment of pseudocysts

**Figure S1**. Sensitivity analysis of Clinical Success

**Figure S2**. Sensitivity analysis of early adverse events

**Figure S3**. Sensitivity analysis of PP recurrence

**Figure S4**. Publication bias analysis for clinical success outcome

**Supporting File 6**: deo270165‐sup‐0006‐SuppMat.docx

**Supporting File 7**: deo270165‐sup‐0007‐SuppMat.docx
