# Supplementary material for: Endoscopic Ultrasound‐guided Drainage With Lumen‐apposing Metal Stent versus Plastic Stent for the Treatment of Pancreatic Pseudocyst: A Systematic Review and Meta‐analysis
Source: DEN Open. 2025 Jun 22;6(1):e70165. doi: 10.1002/deo2.70165 (PMC12182979; doi:10.1002/deo2.70165)
Supplement: Supplementary file 1 — Table S1. Full search strategy for each database Table S2. Risk of bias and quality of studies Table S3. Types of early adverse events of DPPS versus LAMS on the treatment of pseudocysts Table S4. Comparison between the two methods (LAMS and DPPS) for each type of early adverse event Table S5. Types of late adverse events of DPPS versus LAMS on the treatment of pseudocysts [file DEO2-6-e70165-s003.docx]

**SUPPLEMENTARY MATERIALS**

| Database | Search Query |
| --- | --- |
| LILACS/IBECS | #1- Problem- MH:"Pseudocisto Pancreático" OR (Pseudocisto Pancreático) OR MH:C04.182.640.692$ OR MH:C06.689.500.692$ OR MH:"Cisto Pancreático" OR (Cisto Pancreático) OR MH:C04.182.640$ OR MH:C06.689.500$ #2- Intervention- MH:"Aspiração por Agulha Fina Guiada por Ultrassom Endoscópico" OR (Aspiração por Agulha Fina Guiada por Ultrassom Endoscópico) OR MH:E01.370.225.500.384.100.119.500.500$ OR MH:E01.370.225.500.384.100.370.500$ OR MH:E01.370.225.998.054.119.500.500$ OR MH:E01.370.225.998.054.370.500$ OR MH:E01.370.350.850.855.500$ OR MH:E01.370.388.100.100.500.500$ OR MH:E01.370.388.100.370.500$ OR MH:E04.074.119.500.500$ OR MH:E04.074.370.500$ OR MH:E04.502.890.500$ OR MH:E05.200.500.384.100.119.500.500$ OR MH:E05.200.500.384.100.370.500$ OR MH:E05.200.998.054.119.500.500$ OR MH:E05.200.998.054.370.500$ OR MH:E05.242.384.100.119.500.500$ OR MH:E05.242.384.100.370.500$ OR MH:Endossonografia OR Ecoendoscopia OR (Ecografia Endoscópica) OR (Endoscopia Ultrassônica) OR (Ultrassonografia Endoscópica) OR MH:E01.370.350.850.280$ |
| MEDLINE | ("Pancreatic Pseudocyst"[Mesh] OR Pancreatic Pseudocysts OR Pseudocyst, Pancreatic OR Pseudocysts, Pancreatic OR "Pancreatic Cyst" [Mesh] OR Cyst, Pancreatic OR Cysts, Pancreatic OR Pancreatic Cysts OR Pancreatic fluid collections) AND ("Endosonography" [Mesh] OR Endosonographies OR Endoscopy, Echo OR Echo Endoscopies OR Endoscopies, Echo OR Echo-Endoscopy OR Echo-Endoscopies OR Echo Endoscopy OR Endoscopy, Ultrasonic OR Endoscopies, Ultrasonic OR Ultrasonic Endoscopies OR Ultrasonic Endoscopy OR Ultrasonography, Endoscopic OR Endoscopic Ultrasonography OR Endoscopic Ultrasonographies OR Ultrasonographies, Endoscopic OR EUS-guided drainage). |
| EMBASE | #1 Problem 'pancreas cyst'/exp OR 'cyst, pancreas' OR 'pancreas giant cyst' OR 'pancreatic cyst' OR 'polycystic pancreas' OR 'pancreas cyst'   #2 Intervention 'endoscopic ultrasonography'/exp OR 'echoendoscopy' OR 'echography, endoscopic' OR 'endoscopic echography' OR 'endoscopic ultrasound' OR 'endosonography' OR 'EUS (endoscopic ultrasound)' OR 'endoscopic ultrasonography'   3# #1 AND #2   4# #3 AND [embase]/lim NOT ([embase]/lim AND [medline]/lim) |
| Cochrane Library | (Pancreatic Pseudocyst OR Pancreatic Pseudocysts OR Pseudocyst, Pancreatic OR Pseudocysts, Pancreatic OR Pancreatic Cyst OR Cyst, Pancreatic OR Cysts, Pancreatic OR Pancreatic Cysts OR Pancreatic fluid collections) AND (Endosonography OR Endosonographies OR Endoscopy, Echo OR Echo Endoscopies OR Endoscopies, Echo OR Echo-Endoscopy OR Echo-Endoscopies OR Echo Endoscopy OR Endoscopy, Ultrasonic OR Endoscopies, Ultrasonic OR Ultrasonic Endoscopies OR Ultrasonic Endoscopy OR Ultrasonography, Endoscopic OR Endoscopic Ultrasonography OR Endoscopic Ultrasonographies OR Ultrasonographies, Endoscopic OR EUS-guided drainage). |

**Supplementary Table 1:** Full search strategy for each database

**SUPPLEMENTARY MATERIALS**

| Study | Year | D1 | D2 | D3 | D4 | D5 | D6 | D7 | Overall |
| --- | --- | --- | --- | --- | --- | --- | --- | --- | --- |
| Khodakaram K | 2024 | Low risk of bias except for concerns about residual confounding | Low Risk of Bias | Some Concerns | Low Risk of Bias | Low Risk of Bias | Low Risk of Bias | Low Risk of Bias | Some Concerns |
| Gkolfakis P | 2022 | Low risk of bias except for concerns about residual confounding | Low Risk of Bias | Some Concerns | Low Risk of Bias | Low Risk of Bias | Low Risk of Bias | Low Risk of Bias | Some Concerns |
| Kayal A | 2021 | Low risk of bias except for concerns about residual confounding | Low Risk of Bias | Some Concerns | Low Risk of Bias | Low Risk of Bias | Low Risk of Bias | Low Risk of Bias | Some Concerns |
| Al Lehibi A | 2021 | Low risk of bias except for concerns about residual confounding | Low Risk of Bias | Some Concerns | Low Risk of Bias | Low Risk of Bias | Low Risk of Bias | Low Risk of Bias | Some Concerns |
| Yang J | 2019 | Low risk of bias except for concerns about residual confounding | Low Risk of Bias | Some Concerns | Low Risk of Bias | Some Concerns | Low Risk of Bias | Low Risk of Bias | Some Concerns |
| Shin HC | 2019 | Low risk of bias except for concerns about residual confounding | Low Risk of Bias | Some Concerns | Low Risk of Bias | Some Concerns | Low Risk of Bias | Low Risk of Bias | Some Concerns |
| Cho CM | 2018 | High Risk of Bias | Low Risk of Bias | Some Concerns | Low Risk of Bias | Some Concerns | Low Risk of Bias | Low Risk of Bias | High Risk of Bias |
| Ge N | 2017 | Low risk of bias except for concerns about residual confounding | Low Risk of Bias | Some Concerns | Low Risk of Bias | Low Risk of Bias | Low Risk of Bias | Low Risk of Bias | Some Concerns |
| Bang JY | 2017 | Low risk of bias except for concerns about residual confounding | Low Risk of Bias | Some Concerns | Low Risk of Bias | Low Risk of Bias | Low Risk of Bias | Low Risk of Bias | Some Concerns |
| Mukai S. | 2014 | High Risk of Bias | Low Risk of Bias | Some Concerns | Low Risk of Bias | Some Concerns | Low Risk of Bias | Low Risk of Bias | High Risk of Bias |

**Supplementary Table 2.** Risk of bias and quality of studies

Legend: D1- Risk of bias due to confounding/ D2- Risk of bias arising from measurement of exposure/ D3- Risk of bias in selection of participants into the study/ D4- Risk of bias due to post-exposure interventions/ D5- Risk of bias due to missing data/ D6- Risk of bias arising measurement of the outcome/ D7- Risk of bias in selection of the reported result

| **Study** | **Year** | **Events A** | **Total A** | **Events B** | **Total B** | **Bleeding (A)** | **Bleeding (B)** | **Cyst leak (A)** | **Cyst leak**  **(B)** | **Migration/**  **Perfuration (A)** | **Migration/**  **Perfuration (B)** | **Pain (A)** | **Pain (B)** | **Abscess (A)** | **Abscess (B)** | **Infection (A)** | **Infection (B)** | **Other (A)** | **Other (B)** |
| --- | --- | --- | --- | --- | --- | --- | --- | --- | --- | --- | --- | --- | --- | --- | --- | --- | --- | --- | --- |
| **Khodakaram K** | 2024 | 1 | 22 | 2 | 15 | 0 | 1 | 0 | 0 | 1 | 1 | 0 | 0 | 0 | 0 | 0 | 0 | 0 | 0 |
| **Gkolfakis P** | 2022 | 4 | 40 | 7 | 52 | NA | NA | NA | NA | NA | NA | NA | NA | NA | NA | NA | NA | NA | NA |
| **Kayal A** | 2021 | 3 | 11 | 0 | 4 | 2 | 0 | 0 | 0 | 0 | 0 | 1 | 0 | 0 | 0 | 0 | 0 | 0 | 0 |
| **Al Lehibi A** | 2021 | 2 | 10 | 0 | 6 | 2 | 0 | 0 | 0 | 0 | 0 | 0 | 0 | 0 | 0 | 0 | 0 | 0 | 0 |
| **Yang J** | 2019 | 22 | 125 | 6 | 80 | 7 | 0 | 0 | 0 | 2 | 3 | 0 | 0 | 0 | 0 | 11 | 1 | 2 | 2 |
| **Shin HC** | 2019 | 4 | 17 | 2 | 8 | 2 | 0 | 0 | 0 | 2 | 1 | 0 | 1 | 0 | 0 | 0 | 0 | 0 | 0 |
| **Ge N** | 2017 | 1 | 40 | 2 | 12 | 0 | 2 | 1 | 0 | 0 | 0 | 0 | 0 | 0 | 0 | 0 | 0 | 0 | 0 |
| **Mukai S.** | 2014 | 0 | 9 | 1 | 3 | NA | NA | NA | NA | NA | NA | NA | NA | NA | NA | NA | NA | NA | NA |
|  |  |  |  |  |  |  |  |  |  |  |  |  |  |  |  |  |  |  |  |
| **A= DPPS**  **B= LAMS** | |  |  |  |  |  |  |  |  |  |  |  |  |  |  |  |  |  |  |

**Supplementary Table 3.** Types of early adverse events of DPPS versus LAMS on the treatment of pseudocysts

Legend: PP: Pancreatic pseudocyst/ DPPS: Double pigtail plastic stents/ LAMS: lumen-apposing metal stents/ NA: not available

|  |  |  |  |  |  |  |
| --- | --- | --- | --- | --- | --- | --- |
|  | **Number of** | **Total events** | **Total events** |  |  |  |
| **Outcomes** | **studies** | **of DPPS** | **of LAMS** | **RR (95% CI)** | **I2** | **P value** |
| Bleeding | 6 | 13 | 3 | 1.18 (0.27; 5.21) | 35.2% | 0.823 |
| Migration / Perfurantion | 3 | 5 | 5 | 0.60 (0.17; 2.05) | 0.0% | 0.414 |
| Cyst leak | 1 | 1 | 0 | 0.93 (0.04; 21.3) | - | 0.962 |
| Infection | 1 | 11 | 1 | 7.04 (0.93; 53.5) | - | 0.059 |

**Supplementary Table 4.** Comparison between the two methods (LAMS and DPPS) for each type of early adverse event

Legend: DPPS: Double pigtail plastic stents/ LAMS: lumen-apposing metal stents/ RR: Risk Ratio/ I^2^= I-square/ 95% CI: 95% Confidence Interval

| **Study** | **Year** | **Events A** | **Total A** | **Events B** | **Total B** | **Bleeding (A)** | **Bleeding (B)** | **Cyst leak (A)** | **Cyst leak (B)** | **Migration/**  **Perfuration (A)** | **Migration/**  **Perfuration (B)** | **Pain (A)** | **Pain (B)** | **Abscess (A)** | **Abscess (B)** | **Infection (A)** | **Infection (B)** | **Other (A)** | **Other (B)** |
| --- | --- | --- | --- | --- | --- | --- | --- | --- | --- | --- | --- | --- | --- | --- | --- | --- | --- | --- | --- |
| **Kayal A** | 2021 | 3 | 11 | 0 | 4 | 0 | 0 | 0 | 0 | 2 | 0 | 0 | 0 | 1 | 0 | 0 | 0 | 0 | 0 |
| **Ge N** | 2017 | 1 | 40 | 0 | 12 | 0 | 0 | 0 | 0 | 1 | 0 | 0 | 0 | 0 | 0 | 0 | 0 | 0 | 0 |
| **Bang JY** | 2017 | 3 | 14 | 0 | 7 | 0 | 0 | 0 | 0 | 0 | 0 | 0 | 0 | 0 | 0 | 3 | 0 | 0 | 0 |
|  |  |  |  |  |  |  |  |  |  |  |  |  |  |  |  |  |  |  |  |
| **A= DPPS**  **B= LAMS** | |  |  |  |  |  |  |  |  |  |  |  |  |  |  |  |  |  |  |

**Supplementary Table 5.** Types of late adverse events of DPPS versus LAMS on the treatment of pseudocysts

Legend: PP: Pancreatic pseudocyst/ DPPS: Double pigtail plastic stents/ LAMS: lumen-apposing metal stents/ NA: not available.
