# Supplementary figures and images for: Endoscopic Ultrasound‐guided Drainage With Lumen‐apposing Metal Stent versus Plastic Stent for the Treatment of Pancreatic Pseudocyst: A Systematic Review and Meta‐analysis
Source: DEN Open. 2025 Jun 22;6(1):e70165. doi: 10.1002/deo2.70165 (PMC12182979; doi:10.1002/deo2.70165)

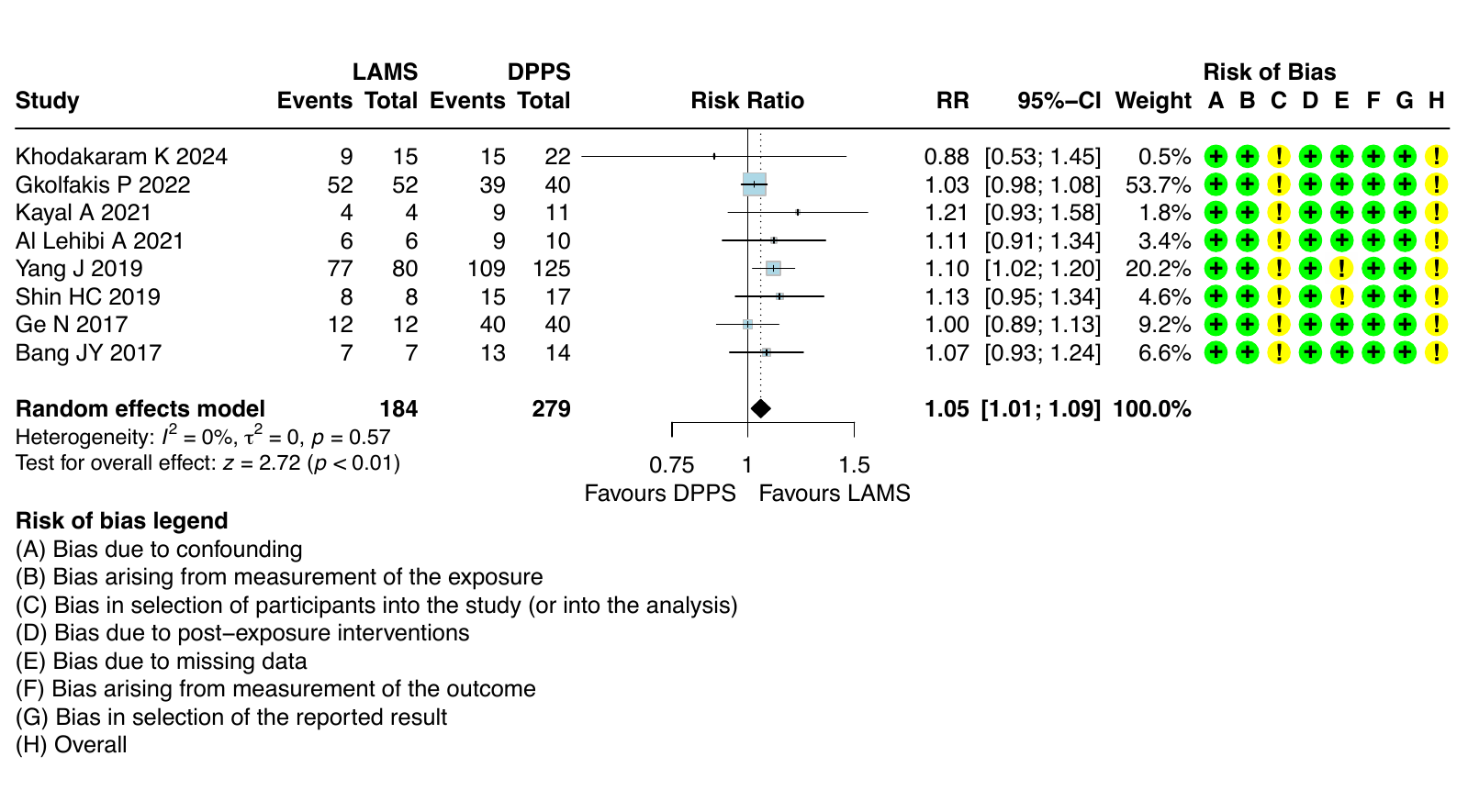

Supplement: Supplementary file 2 — Figure S1. Sensitivity analysis of Clinical Success [file DEO2-6-e70165-s006.tiff]

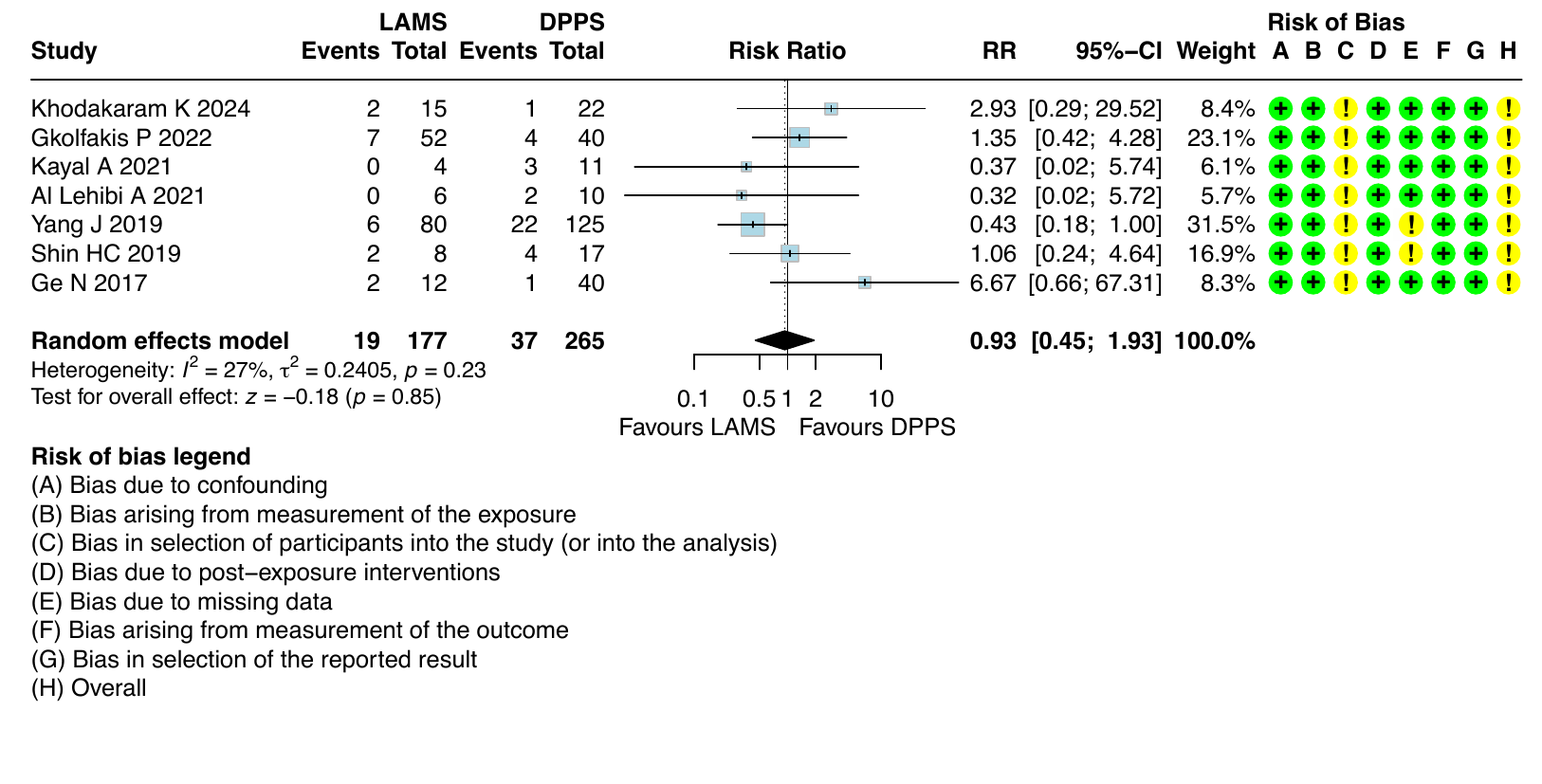

Supplement: Supplementary file 3 — Figure S2. Sensitivity analysis of early adverse events [file DEO2-6-e70165-s002.tiff]

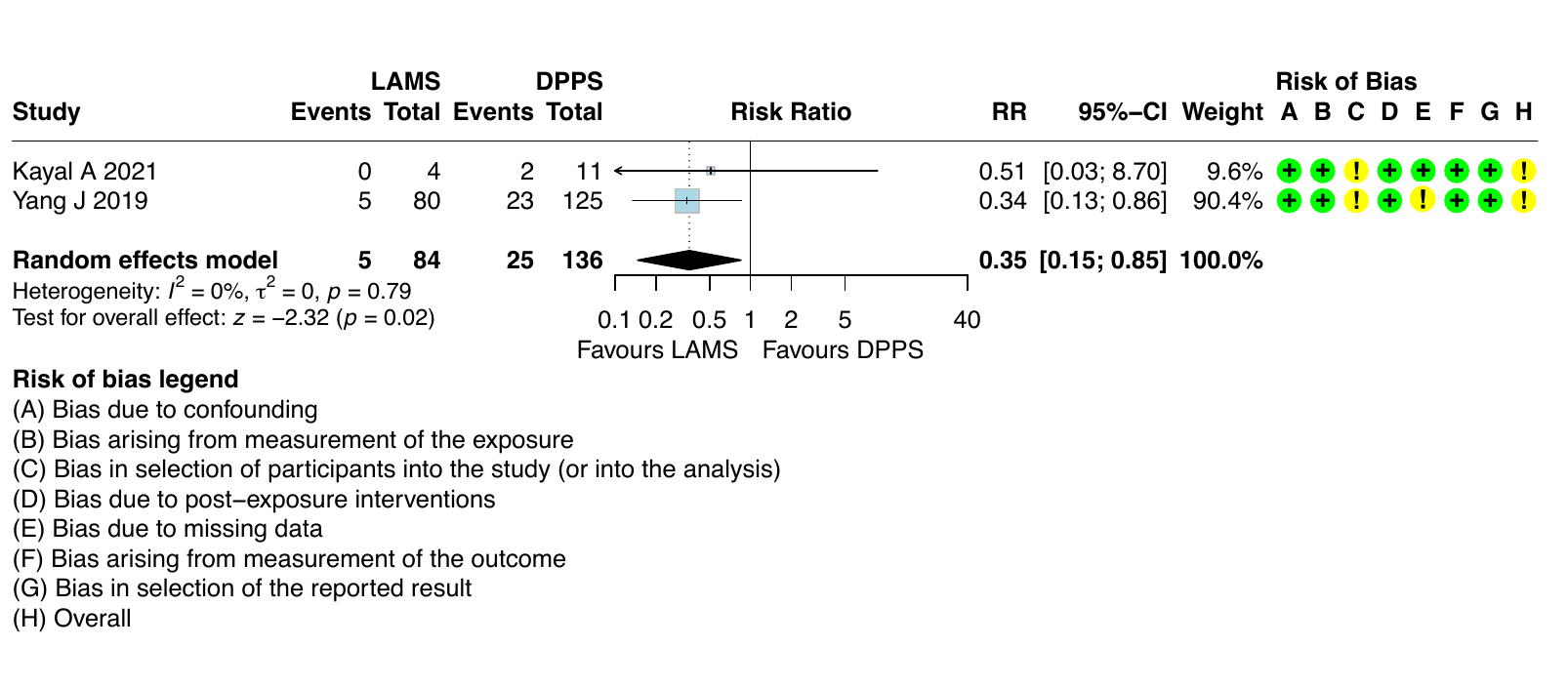

Supplement: Supplementary file 4 — Figure S3. Sensitivity analysis of PP recurrence [file DEO2-6-e70165-s004.tiff]

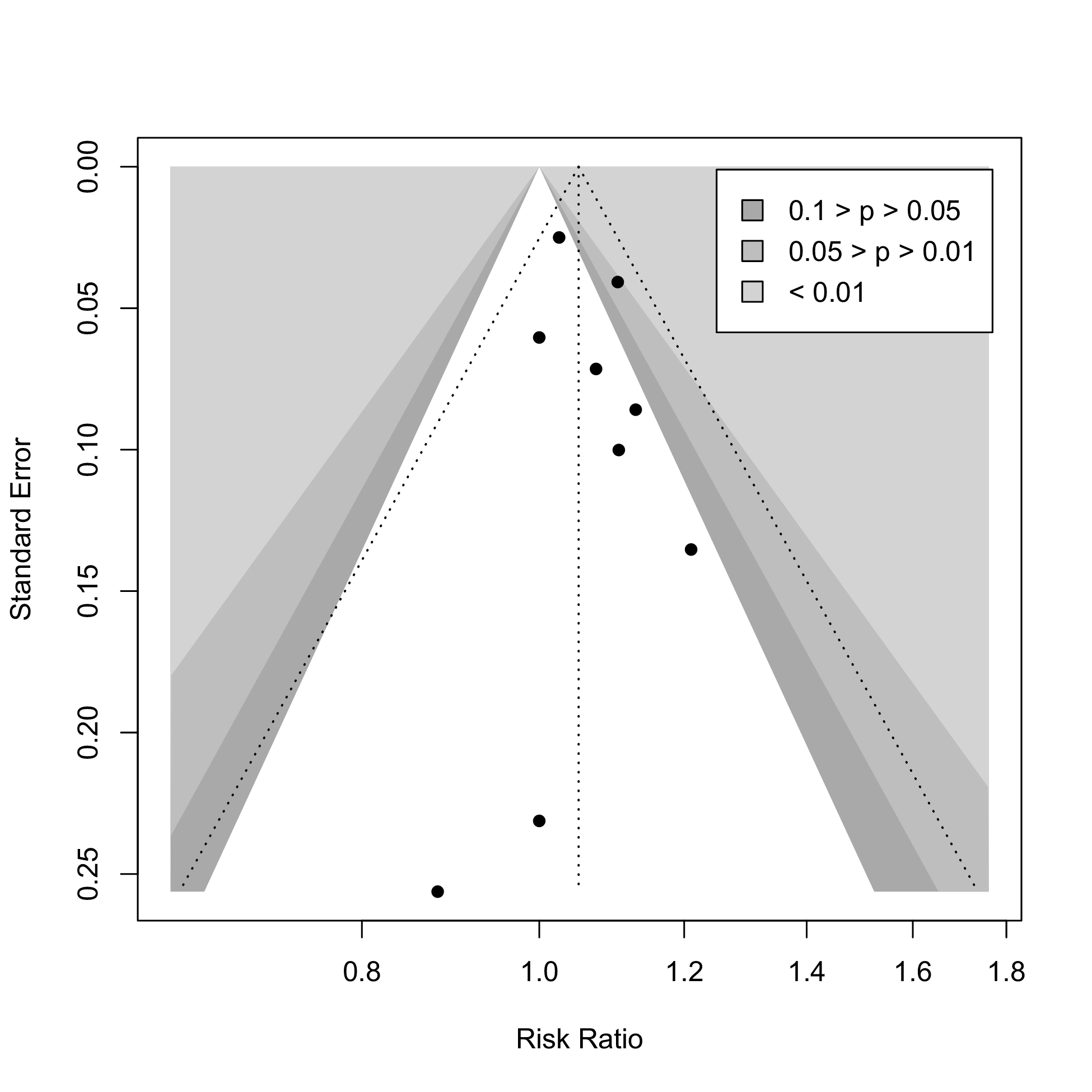

Supplement: Supplementary file 5 — Figure S4. Publication bias analysis for clinical success outcome [file DEO2-6-e70165-s001.tiff]
