# Supplementary material for: Endoscopic Ultrasound‐guided Drainage With Lumen‐apposing Metal Stent versus Plastic Stent for the Treatment of Pancreatic Pseudocyst: A Systematic Review and Meta‐analysis
Source: DEN Open. 2025 Jun 22;6(1):e70165. doi: 10.1002/deo2.70165 (PMC12182979; doi:10.1002/deo2.70165)
Supplement: Supplementary file 6 — Supporting File 6: deo270165‐sup‐0006‐SuppMat.docx [file DEO2-6-e70165-s007.docx]

**SUPPLEMENTARY FIGURE LEGENDS**

**Supplementary Figure 1**. Sensitivity analysis of Clinical Success

**Supplementary Figure 2.** Sensitivity analysis of early adverse events

**Supplementary Figure 3.** Sensitivity analysis of PP recurrence

**Supplementary Figure 4.** Publication bias analysis for clinical success outcome

DPPS: Double pigtail plastic stents/ LAMS: lumen-apposing metal stents/ RR: Risk Ratio/ CI: Confidence interval
