# Supplementary material for: Endoscopic Ultrasound‐guided Drainage With Lumen‐apposing Metal Stent versus Plastic Stent for the Treatment of Pancreatic Pseudocyst: A Systematic Review and Meta‐analysis
Source: DEN Open. 2025 Jun 22;6(1):e70165. doi: 10.1002/deo2.70165 (PMC12182979; doi:10.1002/deo2.70165)
Supplement: Supplementary file 7 — Supporting File 7: deo270165‐sup‐0007‐SuppMat.docx [file DEO2-6-e70165-s005.docx]

**SUPPLEMENTARY TABLE LEGENDS**

**Supplementary Table 1:** Full search strategy for each database

**Supplementary Table 2.** Risk of bias and quality of studies

Legend: D1- Risk of bias due to confounding/ D2- Risk of bias arising from measurement of exposure/ D3- Risk of bias in selection of participants into the study/ D4- Risk of bias due to post-exposure interventions/ D5- Risk of bias due to missing data/ D6- Risk of bias arising measurement of the outcome/ D7- Risk of bias in selection of the reported result

**Supplementary Table 3.** Types of early adverse events of DPPS versus LAMS on the treatment of pseudocysts

Legend: PP: Pancreatic pseudocyst/ DPPS: Double pigtail plastic stents/ LAMS: lumen-apposing metal stents/ NA: not available

**Supplementary Table 4.** Comparison between the two methods (LAMS and DPPS) for each type of early adverse event

Legend: DPPS: Double pigtail plastic stents/ LAMS: lumen-apposing metal stents/ RR: Risk Ratio/ I^2^= I-square/ 95% CI: 95% Confidence Interval

**Supplementary Table 5.** Types of late adverse events of DPPS versus LAMS on the treatment of pseudocysts

Legend: PP: Pancreatic pseudocyst/ DPPS: Double pigtail plastic stents/ LAMS: lumen-apposing metal stents/ NA: not available.
